# Cardiometabolic Risk Among Older Renters and Homeowners

**DOI:** 10.1093/geroni/igaa057.2419

**Published:** 2020-12-16

**Authors:** Sarah Mawhorter, Jennifer Ailshire, Eileen Crimmins

**Affiliations:** 1 University of Southern California, Syracuse, New York, United States; 2 University of Southern California, Los Angeles, California, United States

## Abstract

Researchers consistently find adverse long-term health outcomes among renters as compared with homeowners, yet more proximal health measures are needed to understand whether there is a direct link between tenure and health. In this paper, we compare cardiometabolic risk (CMR) levels among older renters and homeowners, and ask whether this health disparity can be explained by socioeconomic differences between renters and homeowners, or poor housing conditions for renters. Using Health and Retirement Study 2010/2012 biomarker data for adults aged 50-84 (N=10,480), we measure CMR by a scale of C-reactive protein, hemoglobin A1C, cholesterol, heart rate, blood pressure, and waist circumference metrics. We find that renters have greater CMR, even accounting for socioeconomic characteristics and health behaviors. Certain housing and neighborhood conditions, such as perceived safety, are associated with CMR. These results suggest potential pathways through which homeownership confers health advantages over renting.

